# Systematic review of the impact of digital health technologies on blood pressure control and treatment adherence in young and middle-aged hypertensive patients

**DOI:** 10.3389/fcvm.2026.1708019

**Published:** 2026-01-27

**Authors:** Rong Niu, Keye Li

**Affiliations:** Emergency Department, Zhejiang Hospital, Hangzhou, China

**Keywords:** digital healthcare, hypertension, medication adherence, systematic review, young and middle-aged population

## Abstract

**Objective:**

The aim of this work is to evaluate the effect of digital intervention technologies on blood pressure management and medication adherence in hypertensive individuals aged 18–59 years and to explore pathways for precise health management.

**Methods:**

Randomized controlled trials (RCTs) published up to April 2025 were systematically searched in PubMed, Embase, Cochrane Library, Web of Science, and four major Chinese databases (China National Knowledge Infrastructure, Chinese Biomedical Literature Database, Wanfang Data, and Vip Journal Integration Platform). The intervention group received digital interventions such as mobile health apps, remote monitoring, or smart wearable devices, while the control group received routine health education. The Cochrane Risk of Bias tool was used for literature quality assessment, and data analysis was performed using RevMan 5.4 software.

**Results:**

A total of 12 studies (*n* = 1,879) were finally included. Meta-analysis revealed the following: (1) Systolic blood pressure (SBP) decreased by 2.95 mmHg (WMD = −2.95, 95%CI: −4.22 to −1.69). (2) Diastolic blood pressure (DBP) decreased by 3.34 mmHg (WMD = −3.34, 95%CI: −4.63 to −2.06). (3) Medication adherence significantly improved (MD = 2.39, 95%CI: 0.98–3.79, *P* = 0.0009). Considerable heterogeneity was observed in adherence outcomes (I^²^ = 93%). Subgroup analysis based on intervention duration indicated a significant effect within the 24-week subgroup (MD = 2.75, 95%CI: 0.67–4.83), while the 12-week subgroup did not reach statistical significance (MD = 1.85, 95%CI: −0.82–4.52).

**Conclusion:**

Digital health interventions can improve blood pressure control in young and middle-aged hypertensive patients and show potential to enhance medication adherence. However, the observed high heterogeneity and differential effects based on intervention duration suggest that effectiveness may vary. This study provides evidence-based support for the application of digital tools in chronic disease management, while highlighting the need for more tailored and standardized approaches.

## 1 Introduction

Currently, the prevalence of hypertension in Chinese adults is 25.2% (1), with a significant trend toward younger age groups. Due to factors such as occupational stress and concerns about medication use, younger and middle-aged patients demonstrate significantly lower treatment adherence compared with elderly patients, resulting in low awareness rates (20.8%–38.0%), treatment rates (12.0%–27.3%), and control rates (4.3%–9.1%) (2). Emergency data reveal that over 90% of patients with hypertensive emergencies do not receive inpatient treatment (3), highlighting the challenges of out-of-hospital management. Traditional health education has problems such as insufficient standardization of implementation and limited coverage cycles (4). Digital health technologies, by overcoming time and space constraints, create a new path for building precise intervention models. This study aims to evaluate the intervention value of digital technologies in specific populations through evidence-based methods.

## 2 Materials and methods

### 2.1 Inclusion criteria

(1) Study subjects: Individuals aged 18–59 years who met the diagnostic criteria of the Chinese Guidelines for the Prevention and Treatment of Hypertension (2018) [i.e., systolic blood pressure (SBP) ≥140 mmHg and/or diastolic blood pressure (DBP) ≥90 mmHg] were selected. (2) Intervention measures: The intervention group used digital health technologies (mobile health apps, remote monitoring, smart wearable devices, etc.), while the control group received routine health education. (3) Outcome indicators: SBP, DBP, and medication adherence (assessed by scales such as MASES-SF and Hill—Bone Scale) were used as indicators. (4) Study type: RCTs were utilized.

### 2.2 Search strategy

The following electronic databases were systematically searched from their inception to April 2025: PubMed, Embase, Cochrane Library, Web of Science, and four major Chinese databases (China National Knowledge Infrastructure, Chinese Biomedical Literature Database, Wanfang Data, and Vip Journal Integration Platform). The search terms used included the following: (hypertension OR “high blood pressure”) AND (mHealth OR “digital intervention” OR telemedicine) AND (adherence OR compliance). In addition, the references of relevant articles were manually traced to identify any further eligible studies.

### 2.3 Quality assessment

Two researchers independently conducted the following: (1) literature screening and data extraction; (2) methodological quality assessment using the Cochrane Risk of Bias tool (4); and (3) disagreements were resolved through third-party arbitration. Quality grading was defined such that Grade A (low risk of bias) required meeting all six criteria.

### 2.4 Statistical analysis

RevMan 5.4 software was used for all statistical analyses: (1) continuous variables expressed as weighted mean difference (WMD) or standardized mean difference (SMD); (2) heterogeneity testing (random-effects model was applied when I^²^ ≥ 50%); (3) publication bias assessment (Egger’s test); and (4) sensitivity analysis to verify the robustness of conclusions.

## 3 Results

### 3.1 Literature screening process

**Figure 1:**
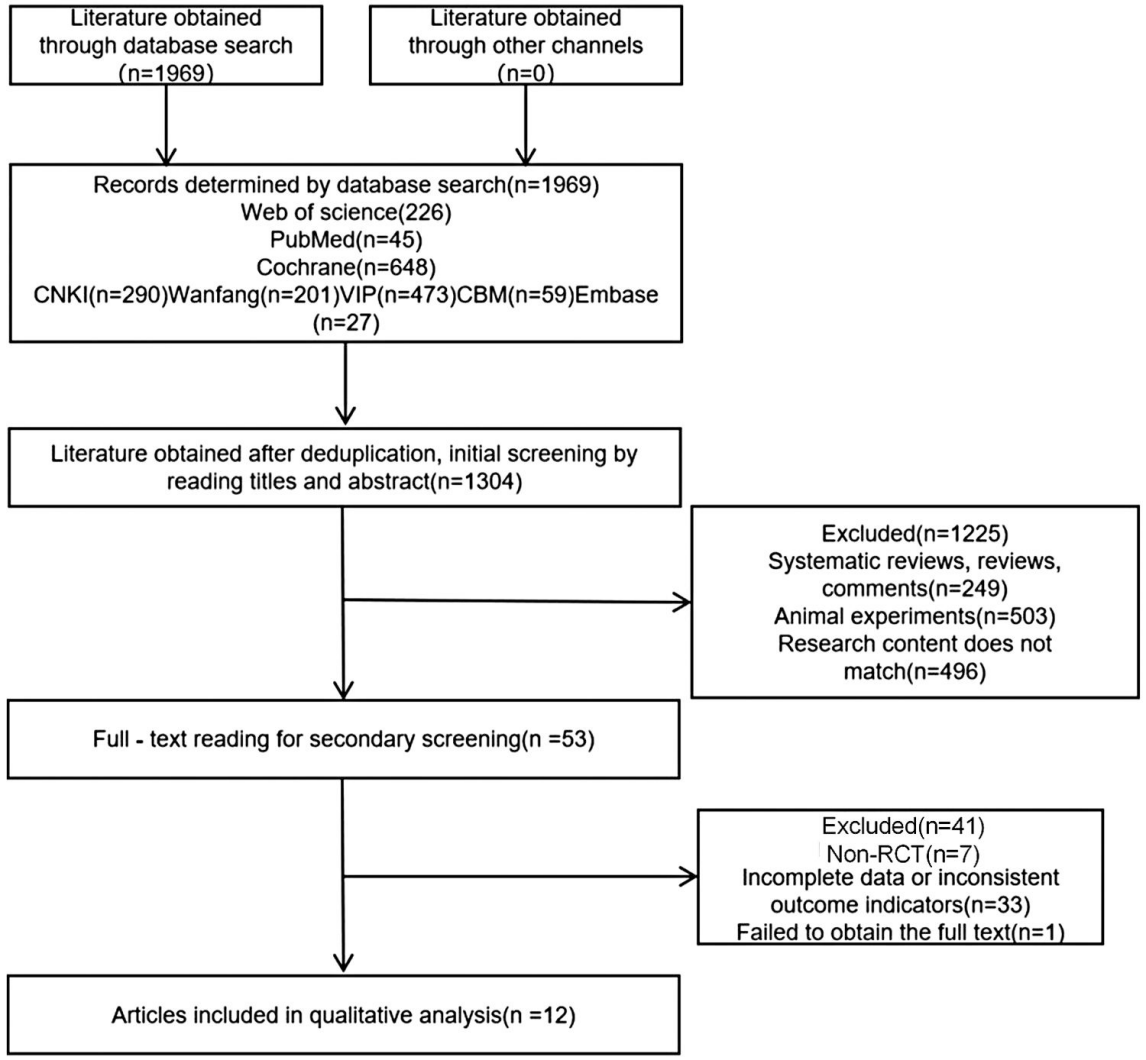
Flowchart of literature screening.

A total of 1,969 articles were initially retrieved. After deduplication, title and abstract screening, and full-text evaluation, 12 RCTs (5–16) were finally included, involving 1,879 patients (938 in the intervention group and 941 in the control group). The basic characteristics of the included studies are presented in Table 1 and Figure 1.

**Table 1:** Characteristics of included studies.

First author	Publication year	Sample size (intervention group/control group)	Baseline blood pressure (mmHg)—intervention group (SBP/DBP)	Baseline blood pressure (mmHg)—control group (SBP/DBP)	Intervention—intervention group	Intervention—control group	Intervention period (weeks)	Outcome indicators
Arabaci (6)	2025	41/41	132.39 ± 13.09/84.15 ± 10.79	129.76 ± 13.69/81.27 ± 10.3	HiperDostum smartphone application (based on the health belief model, including education modules, medication plans, medication reminder systems, and bi-weekly notifications)	Routine medical services	12	Medication adherence self-efficacy scale (MASES-SF), systolic blood pressure (SBP), diastolic blood pressure (DBP)
Bhandari (7)	2022	100/100	134 ± 19.5/84 ± 1.6	137 ± 25.3/86 ± 13.4	Short message intervention (three times a week for 3 months)	Routine care	12	Adherence indicators (Hill Bone Scale), medication adherence self-efficacy, hypertension knowledge score, systolic blood pressure, diastolic blood pressure
Still (9)	2020	30/30	138.51/81.97	138.51/85.28	Online education + home blood pressure monitoring + medication management App + nurse consultation	Enhanced routine care (including home blood pressure monitoring)	12	Medication adherence (Hill–Bone Scale), systolic blood pressure, diastolic blood pressure
Sun (5)	2024	23/31	135.43 ± 17.48/78.39 ± 8.81	136.94 ± 18.44/76.03 ± 9.20	WeChat-based digital intervention (health education + behavior change techniques)	Routine health education + self-management manual	12	Exercise time (MET—min/week), medication adherence score, blood pressure monitoring frequency (% daily monitoring), body weight (kg), SEVRa, learning performance score, systolic blood pressure, diastolic blood pressure
Yuting (8)	2023	66/68	152.59 ± 23.44/92.85 ± 14.94	148.85 ± 20.70/91.34 ± 15.31	Wearable blood pressure monitoring device + smartphone APP (reminders, medication reports, medical guidance, family support)	Routine community management	12	Waist circumference, hypertension adherence, self-efficacy, physical health, systolic blood pressure, diastolic blood pressure
Xueying (10)	2024	33/34	130.91 ± 7.80/83.21 ± 6.6	133.21 ± 5.87/83.30 ± 4.19	Routine medication care + medication literacy promotion program	Routine medication care	12	Medication literacy level, medication adherence, medication self-efficacy, systolic blood pressure, diastolic blood pressure
Qing (11)	2019	100/100	152.01 ± 12.13/93.17 ± 7.37	153.26 ± 13.06/92.41 ± 8.46	WeChat platform-extended nursing intervention based on attribution theory	Routine community care under the family doctor contract model	24	Systolic blood pressure, diastolic blood pressure, self-management, medication adherence
Na (12)	2023	56/56	150.13 ± 10.724/92.472 ± 3.262	150.11 ± 10.547/93.056 ± 3.853	Intervention program based on the Cox health behavior interaction model (health lectures, mobile phone push, remote counseling, hands-on experience, telephone interaction, patient exchange meetings, WeChat follow-up, etc.)	Routine health education intervention	24	BMI, total self-management score, self-efficacy score, medication adherence score, blood pressure control effectiveness rate, systolic blood pressure, diastolic blood pressure
Huicai (13)	2019	131/131	144.56 ± 17.98/93.58 ± 13.65	144.14 ± 15.94/90.44 ± 11.26	O2O model (WeChat platform + offline guidance)	Community contract-based services	24	Systolic blood pressure, diastolic blood pressure, hypertension-related knowledge
					Online WeChat platform health education + offline home visits and health education lectures			
Lijuan (14)	2020	50/50	134.86 ± 8.24/83.64 ± 5.65	135.98 ± 11.42/82.64 ± 4.06	Routine community chronic management	Routine community chronic management (stated as control group in the original text, organized as per the figure)	24	Systolic blood pressure, diastolic blood pressure, medication adherence, blood pressure control rate, hypertension knowledge awareness rate, self-management behavior
Lijuan (15)	2018	100/100	149.65 ± 16.78/95.72 ± 6.82	148.87 ± 17.87/96.71 ± 5.98	Complication simulation experience education + routine education	Routine education	4	Systolic blood pressure, diastolic blood pressure, medication adherence, ATRABS level
								Hypertension knowledge score (total score 22 points), treatment adherence (total 112 points)
Bei (16)	2018	115/111	144.56 ± 17.98/93.58 ± 13.65	1441. 4 ± 15.94/90.44 ± 11.26	WeChat-based mobile medical intervention (weekly health education content push + online consultation + offline appointment)	Community contract-based services (health records + health education lectures + telephone follow-up)	24	Systolic blood pressure, diastolic blood pressure, BMI (kg/m^²^), medication adherence (total 112 points)

### 3.2 Characteristics of included studies

### 3.2.1 Quality assessment

**Figure 2:**
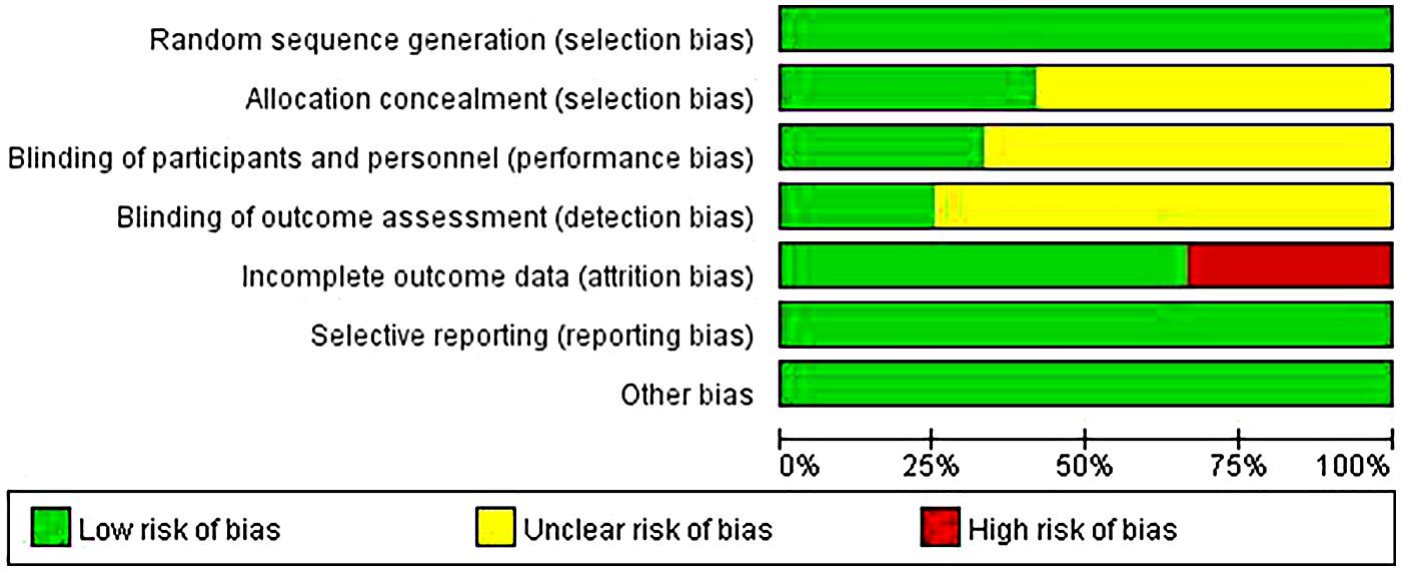
Bar chart of risk of bias for included studies. Bar chart generated after methodological quality assessment of 12 included studies using the Cochrane risk of bias tool.

The quality of included literature: Three studies were Grade A (6, 7, 14) (low risk of bias) and nine studies were Grade B (5, 8, 13, 15) (moderate risk of bias). The main sources of bias were insufficient allocation concealment and lack of blinding, as shown in Table 2 and Figure 2.

**Table 2:** Methodological quality assessment of included literature.

First author	Randomization	Allocation concealment	Blinding	Blinding	Completeness of result data	Selective reporting of research results	Other bias sources	Literature quality grade
			(Research subject/implementer of intervention)	(Outcome assessor)				
Yang (12)	Random number table method	Concealed allocation sequence	Unclear	Unclear	Five lost to follow-up (two in control group, three in intervention group)	None	None	Grade B
Qing (11)	Random number table method	Not mentioned	Unclear	Unclear	None	None	None	Grade B
Xueying (10)	Computer random number table method	Allocation sequence concealed	Unclear	Yes	Five lost to follow-up (three in intervention group, two in control group)	None	None	Grade B
Arabaci (6)	Randomized controlled trial	Allocation sequence concealed	Yes	Yes	None	None	None	Grade A
Bhandari (7)	Randomized controlled trial	Allocation concealed	Yes	Yes	None	None	None	Grade A
Still (9)	Randomized controlled trial	Not mentioned	Unclear	Unclear	None	None	None	Grade B
Sun (5)	Random number table method	Not mentioned	Unclear	Unclear	14 lost to follow-up	None	None	Grade B
Yuting (8)	Randomized controlled trial	Not mentioned	Unclear	Unclear	14 lost to follow-up	None	None	Grade B
Bei (16)	Computer randomization	Not mentioned	Unclear	Unclear	Not reported	None	None	Grade B
Lichang (15)	Random number table method	Not mentioned	Unclear	Unclear	None	None	None	Grade B
Huicai et al. (13)	Random grouping	Not mentioned	Unclear	Unclear	Not reported	None	None	Grade B
Lijuan (14)	Computer randomization	Allocation concealed	Yes	Yes	None	None	None	Grade A

### 3.3 Results of meta-analysis

**Figure 3:**
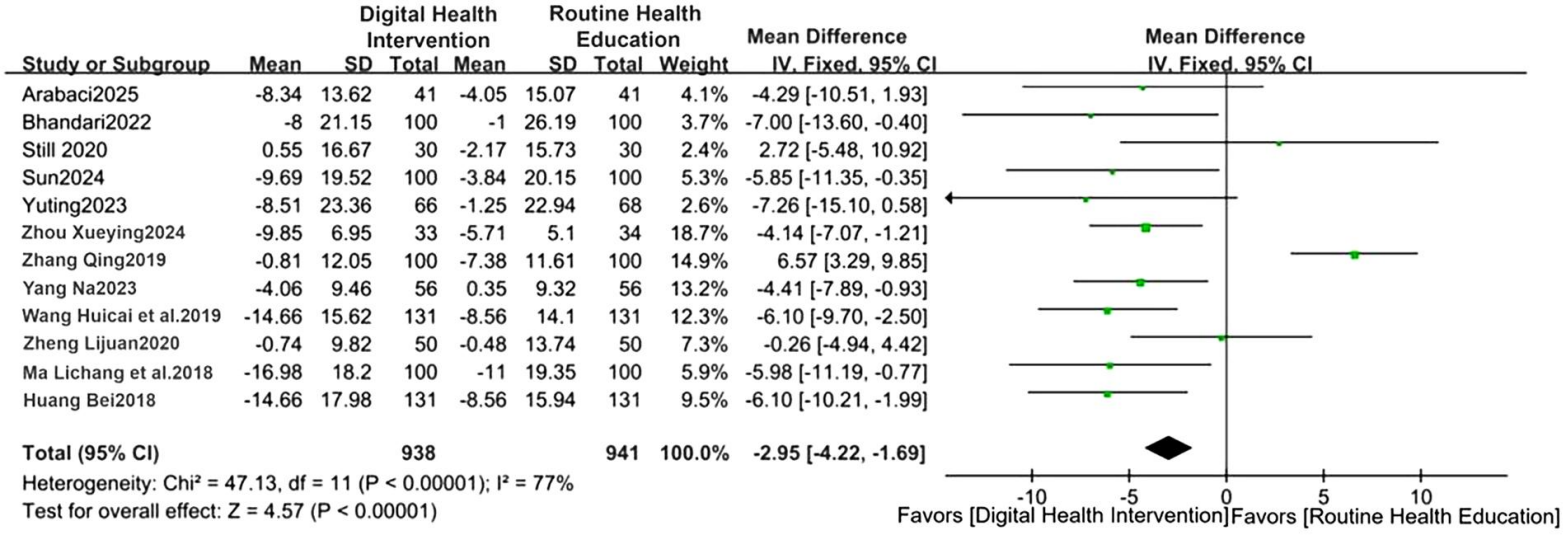
Forest plot of SBP comparison (WMD = −2.95). Forest plot comparing changes in SBP between the digital health intervention group and the routine health education control group via meta-analysis.

**Figure 4:**
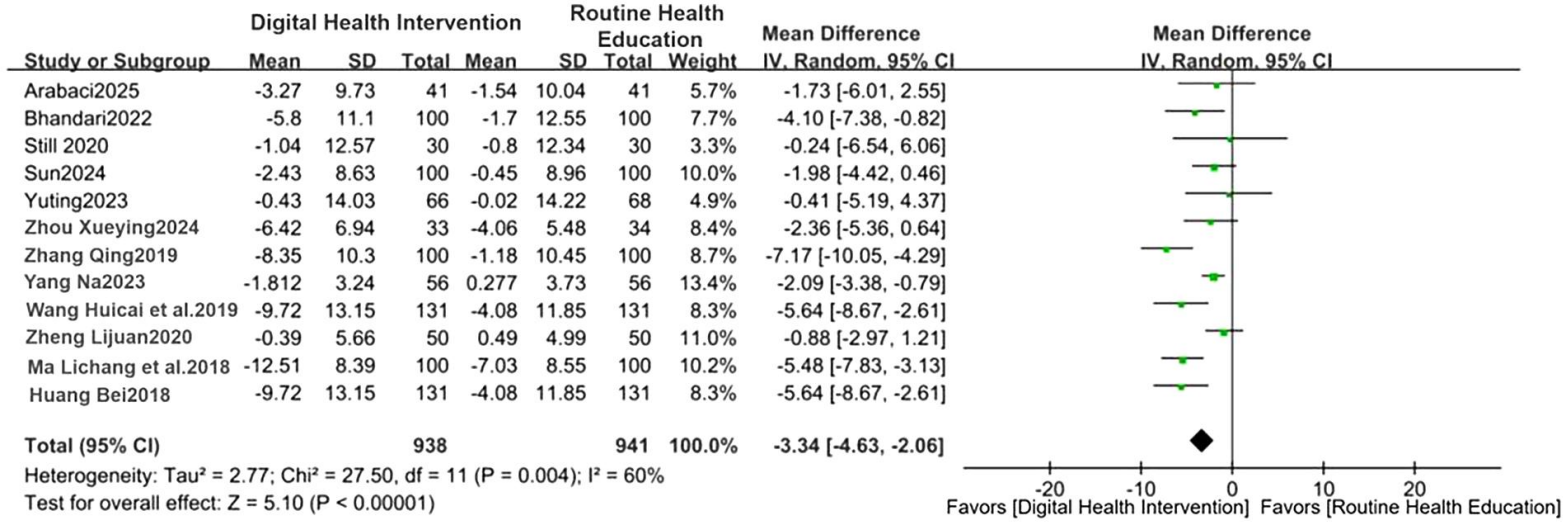
Forest plot of DBP comparison (WMD = −3.34). Forest plot comparing changes in DBP between the digital health intervention group and the routine health education control group via meta-analysis.

**Figure 5:**
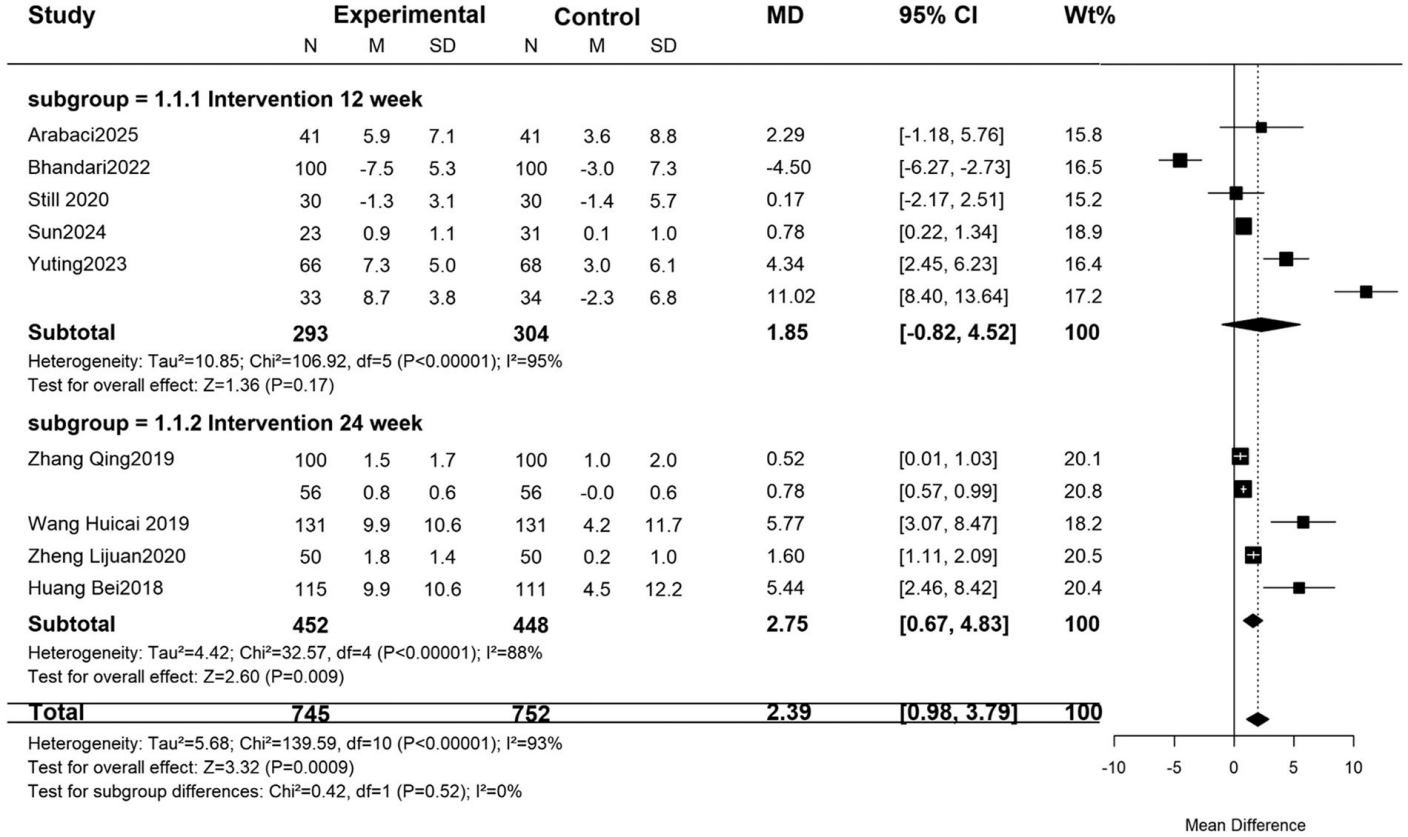
Subgroup analysis of the impact of different intervention durations on adherence. Forest plot comparing the impact of digital health interventions on medication adherence by subgroup analysis of intervention durations (12 and 24 weeks).

Blood pressure control: The digital intervention group showed a decrease in SBP by 2.95 mmHg (95%CI: −4.22 to −1.69, *P* < 0.00001) and DBP by 3.34 mmHg (95%CI: −4.63 to −2.06, *P* = 0.004), with effect sizes shown in Figures 3, 4. For treatment adherence, the digital health interventions group demonstrated a statistically significant improvement compared to the routine health education group (MD = 2.39, 95%CI: 0.98–3.79, *P* = 0.0009; I^²^ = 93%, random-effects model). Given substantial heterogeneity (I^²^ = 93%), subgroup analysis by intervention duration was performed. The 24-week intervention subgroup showed a significant effect (MD = 2.75, 95%CI: 0.67–4.83, *P* = 0.009; I^²^ = 88%), while the 12-week intervention subgroup did not reach statistical significance (MD = 1.85, 95%CI: −0.82–4.52, *P* = 0.17; I^²^ = 95%). No significant difference was observed between subgroups (*P* = 0.52), suggesting that the heterogeneity may be attributed to factors other than intervention duration (Figure 5).

## 4 Discussion

This meta-analysis confirms that digital health interventions exert significant clinical effects on improving blood pressure control in young and middle-aged hypertensive patients. Compared with routine health education, digital health interventions can reduce SBP by an average of 2.95 mmHg (WMD = −2.95, 95%CI: −4.22 to −1.69) and DBP by an average of 3.34 mmHg (WMD = −3.34, 95%CI: −4.63 to −2.06). These findings are consistent with the conclusions of the latest systematic review published in 2024, which also confirmed that digital interventions such as mHealth and telemedicine can significantly reduce SBP and DBP in hypertensive patients (17). However, the magnitude of blood pressure reduction in our study appears more modest compared with some other meta-analyses. For example, a recent meta-analysis focusing specifically on patients with uncontrolled baseline hypertension reported a more pronounced SBP reduction of 4.45 mmHg (18). This discrepancy is likely attributable to differences in study populations; our inclusion of patients with better-controlled baseline blood pressure may have limited the potential for larger reductions. This suggests that the effectiveness of digital interventions is modulated by the initial hypertension severity. It is also worth noting that the present study found a slightly greater reduction in DBP than in SBP. This observation may be linked to the specific vascular physiology of our younger study cohort. In individuals under 50 years, interventions that improve peripheral vascular resistance can exert a more substantial impact on DBP, whereas the age-related increase in large arterial stiffness, which primarily drives SBP, is less pronounced. Therefore, the observed effect may reflect a genuine physiological response to digital health interventions within this demographic (19).

The superiority of digital health interventions in blood pressure control stems from their multi-target mechanisms of action. First, continuous blood pressure monitoring via smart wearable devices addresses the “white-coat effect” and insufficient monitoring of traditional office-based blood pressure measurement, providing comprehensive data support for treatment adjustments (20). Second, personalized feedback systems based on artificial intelligence algorithms can dynamically analyze lifestyle data of patients and provide precise behavioral intervention recommendations, resulting in an average reduction of 14.66 mmHg in SBP and 8.56 mmHg in DBP in Stage 2 hypertensive patients within 24 weeks (16). Third, digital interventions significantly improve the standardization and continuity of treatment through functions such as medication reminders, adherence tracking, and electronic pill boxes (6, 8, 9, 21, 22). Crucially, the innovation of this study lies in its specific focus on young and middle-aged patients, a population for which there is a recognized “guidance gap” in clinical management. As noted in a scientific statement from the American Heart Association, there is a lack of clear recommendations for managing Stage 1 hypertension in lower-risk adults, who are predominantly from this age group (23). Furthermore, young and middle-aged patients face unique self-management challenges, including lower disease awareness and competing work–life pressures (24). Digital health tools are particularly well suited to address these barriers by providing convenient, discreet, and integrated support, thus filling a critical niche in the hypertension care continuum. Clifford et al.’s 's (20) study summarized the current status of precision health research, emphasizing the importance of personalization, phenotyping, and prediction of adult hypertension management using digital health tools. It proposed that more interdisciplinary collaboration and ultimately interdisciplinary approaches are needed to meaningfully advance the field of precision health in hypertension risk prediction, prevention, and management.

The findings of this review should be interpreted in light of the strengths and limitations of the included studies. A key strength is that all synthesized evidence originated from RCTs, which represent the highest level of primary study design for evaluating interventions. Several studies incorporated objective outcome measures, such as automated blood pressure monitoring data, alongside self-reported adherence scales, thereby enhancing the validity of the results. Furthermore, a number of trials were conducted over 24 weeks, providing insights into the medium-term sustainability of digital health effects. However, methodological limitations of the primary studies must be acknowledged. The main sources of bias arose from inadequate allocation concealment and a lack of blinding of participants and personnel, which is a common but notable challenge in behavioral intervention trials. Attrition bias was present in some studies, and several had relatively small sample sizes, limiting the precision of individual findings. The considerable clinical heterogeneity—stemming from variations in the specific digital platforms used, intervention components, and cultural contexts—further complicates direct comparisons. These limitations underscore that while the pooled results are encouraging, they are derived from an evolving evidence base with inherent methodological variabilities.

Despite the positive conclusions of this study, the following limitations should be acknowledged: Larger sample sizes and higher-quality RCTs are still needed to further verify the conclusions of this study. Digital interventions are diverse (apps, wearable devices, telemedicine, etc.), but the differences in effectiveness between different technical forms have not been fully analyzed. The response differences among patients with different cultural backgrounds and educational levels have not been deeply analyzed.

Future research should focus on developing phenotype-oriented intervention matching strategies, dynamic dose adjustment technologies, and social-–ecological integration models, while strengthening long-term effects and economic evaluation. Interventions for young and middle-aged populations need to focus on precise pathway design. By integrating digital technology, precision medicine, and health system reform, we have the potential to reverse the severe situation of the “three lows” (low awareness rate, low treatment rate, and low control rate) in young and middle-aged people with hypertension. Ultimately, this approach may reduce lifelong cardiovascular risk in this critical population and contribute to achieving the “Healthy China 2030” strategic goal.

## Data Availability

The original contributions presented in the study are included in the article/Supplementary Material; further inquiries can be directed to the corresponding author.
